# Temporal Factors Associated With Opioid Prescriptions for Patients With Pain Conditions in an Urban Emergency Department

**DOI:** 10.1001/jamanetworkopen.2020.0802

**Published:** 2020-03-25

**Authors:** Ben C. Smith, Andrew D. Vigotsky, A. Vania Apkarian, Thomas J. Schnitzer

**Affiliations:** 1 Medical Student, Feinberg School of Medicine, Northwestern University, Chicago, Illinois; 2 Department of Biomedical Engineering, Northwestern University, Evanston, Illinois; 3 Department of Statistics, Northwestern University, Evanston, Illinois; 4 Center for Translational Pain Research, Feinberg School of Medicine, Northwestern University, Chicago, Illinois; 5 Anesthesiology and Medicine (Rheumatology), Feinberg School of Medicine, Northwestern University, Chicago, Illinois

## Abstract

**Importance:**

Opioid prescriptions for treatment of pain in emergency departments (EDs) are associated with long-term opioid use. The temporal pattern of opioid prescribing in the context of the opioid epidemic remains unknown.

**Objective:**

To examine the temporal pattern of opioid prescribing within an ED for varying pain conditions between 2009 and 2018.

**Design, Setting, and Participants:**

A population-based, cross-sectional study was conducted at the ED of an urban academic medical center. All patients treated within that ED between January 1, 2009, and December 31, 2018, were included.

**Main Outcomes and Measures:**

The proportion of patients prescribed an opioid for treatment of pain in the ED temporally by condition, condition type, patient demographics, and physician prescriber.

**Results:**

Between 2009 and 2018, 556 176 patient encounters took place in the ED, with 70 218 unique opioid prescriptions ordered. A total of 316 632 patients (55.9%) were female, 45 070 (42.6%) were of white race, and 43 412 (40.6%) were privately insured; the median age group was 41 to 45 years. Yearly opioid prescriptions decreased by 66.3% (from 16.3 to 5.5 opioids per 100 encounters) between 2013 and 2018, with a yearly adjusted odds ratio (aOR) of 0.808 (95% CI, 0.802-0.814) compared with the prior year. In patients with musculoskeletal pain (back, joint, limb, and neck pain), opioid prescribing decreased by 71.1% (from 36.7 to 10.6 opioids per 100 encounters between 2013 and 2018; aOR, 0.758; 95% CI, 0.744-0.773). In patients with musculoskeletal trauma (fracture, sprain, contusion, and injury), opioid prescribing decreased by 58.0% (from 34.2 to 14.8 opioids per 100 encounters; aOR, 0.811; 95% CI, 0.797-0.824). In patients with nonmusculoskeletal pain (abdominal pain, kidney stone, respiratory distress, and pharyngitis) opioid prescribing decreased by 53.7% (from 20.1 to 9.3 opioids per 100 encounters; aOR, 0.850; 95% CI, 0.834-0.868). Between 2009 and 2018, patients who were black (aOR, 0.760; 95% CI, 0.741-0.779) and those who were Asian (aOR, 0.714; 95% CI, 0.665-0.764) had the lowest odds of receiving an opioid compared with other racial/ethnic groups.

**Conclusions and Relevance:**

There was a substantial temporal decrease in the number of opioid prescriptions within this ED during the study period. This decrease was associated with substantial relative reductions in opioid prescribing for treatment of musculoskeletal pain compared with fractures and kidney stones.

## Key Points

**Question:**

Have emergency department clinicians responded to the opioid epidemic through altering opioid prescription rates?

**Findings:**

In this cross-sectional study of 556 176 emergency department patient encounters and 70 218 opioid prescriptions within a single emergency department, yearly prescriptions decreased by 66.3% between 2013 and 2018. This decrease was associated with a 71.1% reduction in the number of opioid prescriptions for musculoskeletal pain (back, limb, joint, and neck pain) and lesser, but still marked, decreases for fractures and kidney stones.

**Meaning:**

Reductions in yearly opioid prescriptions across varying indications appear to be aligned with recognition of the opioid crisis in addition to national, state, and departmental education guidelines.

## Teaser

This cross-sectional study examines the temporal pattern of opioid prescribing for patients with pain conditions in an urban emergency department over a 10-year period.

## Introduction

Heightened attention to the prescription of opioids for the treatment of pain has been a central goal in medicine over the past decade. Opioid misuse was associated with 68% of US drug overdose deaths in 2017 and more than 400 000 deaths from 1999 to 2017.^1,2^ In addition, the opioid epidemic has imparted a $631 billion burden to the US economy from 2015 to 2018.^3^ The contribution of emergency medicine to the opioid epidemic has been has been subject to a range of debate from making a minor contribution to the ongoing opioid epidemic^4^ to acting as an origin for repeated use and potential opioid use disorder.^5,6,7^ A 2018 study^8^ suggested that emergency department (ED) prescriptions following new Centers for Disease Control and Prevention guidelines^9^ show little association with long-term opioid use, although up to 13.4% of Medicare patients in the study went on to receive long-term opioid therapy. In any case, a 2015 study reported that 17.1% of all ED patients were discharged with an opioid prescription during the week of data collection,^10^ and a 2017 study demonstrated equal efficacy for certain pain treatment in the ED with nonopioid analgesics.^11^ It is challenging for prescribers to discern the benefits and risks of opioid prescribing within an encounter for acute pain,^12,13,14,15,16^ but with up to two-thirds of all ED patients seeking treatment for pain,^17,18,19^ a 22.2% nationwide reduction in all opioid prescriptions ordered from 2013 to 2017,^20^ and guidelines recommending judicious opioid prescribing,^9,21^ it is important to discern whether emergency medicine is reducing opioid prescribing for the treatment of pain.

The aim of this study was to evaluate temporal changes in overall opioid prescribing and prescriptions for specific pain conditions in an urban academic ED between 2009 and 2018. In addition, the temporal pattern of opioid prescribing at the individual clinician level was examined, as previous studies have indicated that the decrease in opioid prescription counts may be dependent on a subset of clinicians decreasing opioid prescribing, while others maintain high-intensity prescribing, regardless of specialty^22^ and including ED clinicians.^5,23,24^ We also examined demographic factors that may be associated with opioid prescribing to assess the possibility of underlying opioid prescription bias within the ED.

## Methods

All patient encounters in the Northwestern Memorial Hospital ED and Northwestern Memorial Hospital Feinberg Mezzanine Emergency Room, Chicago, Illinois, between January 1, 2009, and December 31, 2018, were selected from the Northwestern Medicine Enterprise Data Warehouse. An encounter was defined by a unique patient (identified by a unique patient identifier) having a unique time and date entered into the Enterprise Data Warehouse database from the electronic health record. An encounter included the self-identified age, sex, race/ethnicity, payer status, opioid prescribed, deidentified physician prescriber, and *International Classification of Diseases, Ninth Revision* (*ICD-9*), and *International Statistical Classification of Diseases, 10th Revision* (*ICD-10*), diagnosis codes for each patient. To fully anonymize the data, the Enterprise Data Warehouse assigned each patient and physician a randomized unique identifier, had visit dates shifted within a 10-day window, and grouped patient age within 5 years to properly deidentify the data set. This study follows the Strengthening the Reporting of Observational Studies in Epidemiology (STROBE) reporting guideline for cross-sectional studies. Exclusion criteria included any encounter without an *ICD* diagnosis and encounters not labeled as emergency. The study was approved by the institutional review board at Northwestern University. All data were deidentified and a waiver of informed consent was granted by the institutional review board.

Opioid prescriptions were manually selected by name of the drug and are included in eTable 1 in the Supplement. Hydrocodone plus acetaminophen was the primary agent, representing 97.1% of all of the prescriptions. Diagnostic conditions were defined using *ICD-9* and *ICD-10* codes and are presented in eTable 2 in the Supplement.

**Figure 1.**
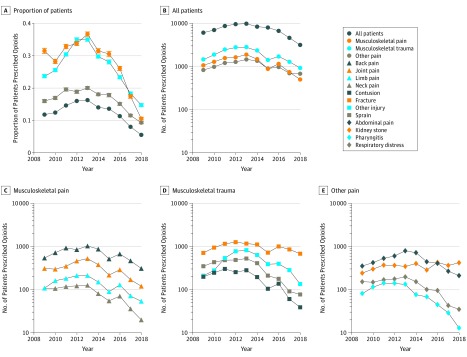
Temporal Opioid Prescribing Within Diagnosis Groups A, Temporal opioid prescriptions within condition groups. B through E, Temporal opioid prescriptions by condition as part of all emergency department opioid prescriptions.

Twelve diagnostic conditions—back pain, joint pain, limb pain, neck pain, fracture, sprain, contusion, other unspecified injury, abdominal pain, kidney stone, respiratory distress, and pharyngitis—were selected for analyses because they had the highest opioid prescription volume. Patients with these conditions accounted for 59.4% of all opioids prescribed and allowed for distinct and convenient grouping of patients based on pain sources (Figure 1). Encounters from 2009 to 2014 had an *ICD-9* code defined as primary, identifying the likely condition for which an opioid was prescribed within the encounter. After 2014, *ICD-10* codes were implemented and primary codes were no longer delineated within the data set obtained. To ensure that the opioid was given for the specific condition, patients within a singular *ICD-10* code were included for selection into a condition. Although data on certain patients may be lost using this criterion, yearly patient counts in each condition remained relatively consistent with the years using *ICD-9* coding, demonstrating few exclusions. Patients with multiple *ICD-10* codes within the same diagnostic group only, most notably fractures, were also included. Any patients with an *ICD-10* code for other unspecified injury were included within this diagnostic group, as this was likely a secondary code in the *ICD-10* system and kept yearly patient counts similar to *ICD-9* years. Because the aim was to look at changes over time, changes from 2009 to 2014 will have consistency within the *ICD-9* system, and those from 2015 to 2018 will have consistency within the *ICD-10* system. From these conditions, patients were categorized into 3 groups: musculoskeletal pain (back, joint, limb, and neck pain), musculoskeletal trauma (fracture, sprain, contusion, and other unspecified injury), and nonmusculoskeletal pain (abdominal pain, kidney stone, respiratory distress, and pharyngitis). These groupings define the source of the pain, identify the observation of objective pathologic factors by the clinician (pain vs trauma), and delineate opioid prescriptions between musculoskeletal and nonmusculoskeletal conditions. Any patient with a fracture, sprain, and/or contusion *ICD-10* code in addition to an other unspecified injury diagnosis code was not double counted in the musculoskeletal trauma grouping.

### Statistical Analysis

Baseline demographic characteristics and characteristics of patient subsets were determined using descriptive analyses. Absolute and relative opioid prescription changes were descriptively evaluated as a function of time, condition group (ie, musculoskeletal trauma, musculoskeletal pain, and other pain), and conditions within condition groups. Proportions and their SEs were calculated with normal approximations (ie, SE = [*p*(1 − *p*)/*n*]^1/2^). Following descriptive evaluation of the data, 2013 was chosen as the reference year for continuous and controlled estimates of the effects of time in our population because that is when opioid prescribing peaked. Opioid prescription counts were determined by sex (male, female), race/ethnicity (white, black, Hispanic, Asian, and other), insurance status (private, Medicare, Medicaid, and self-pay), and age (0-15,16-30, 31-65, and >65 years) for all encounters and in conditions of interest.

Following descriptive evaluation of the data, inferential statistics were carried out to further examine temporal opioid prescribing. Specifically, univariable and multivariable logistic regression models were constructed, with each modeling whether an opioid was prescribed within an encounter as the dependent variable and year as the primary independent variable. Multivariable models incorporated adjustments for age, sex, race/ethnicity, and insurance status (stratified as described in the Methods section); age 31 to 65 years, male, white race, and private insurance were chosen as reference categories because they represented the highest proportion of opioid prescriptions among the patient subgroups. Odds ratios (ORs), adjusted ORs (aORs), and their 95% CIs were calculated. All ORs and aORs represent the odds of receiving an opioid relative to the prior year, with 2013 being the intercept. In addition, a multivariable logistic regression with interaction terms between year and race/ethnicity, year and age, year and sex, and year and insurance status were used to examine whether differences in the reduction of opioid prescriptions from 2013 to 2018 existed within patient subgroups.

Twelve clinicians were selected for having more than 10 000 encounters from 2009 to 2017. Clinician-level data—but not other data—from 2018 were not available, so this year was excluded for clinician-level analyses. These 12 clinicians were chosen because they represented the upper tercile of ED prescribers by opioid prescription numbers during this period and saw a representative caseload in a year over most years, allowing for temporal analysis. Multivariable logistic regression models, which adjusted for patient age, sex, race/ethnicity, and insurance status, were used for individual clinicians to examine their opioid prescribing over time while controlling for patient demographic characteristics. Adjusted ORs and 95% CIs for each clinician were calculated. All data were processed using SAS, version 9.4 (SAS Institute Inc). Findings were considered significant at 2-sided, 2-tailed *P* = .05.

## Results

Between 2009 and 2018, there were 556 176 patient encounters in the ED, with 70 218 unique opioid prescriptions ordered within those encounters. A total of 316 632 (55.9%) patients were female, 45 070 (42.6%) were of white race, and 43 412 (40.6%) were privately insured; the median age group was 41 to 45 years. Of patients who did not receive an opioid, 316 632 (56.1%) were female, 245 070 (41.9%) were white, and 143 412 (39.8%) were privately insured; the median age group was 41 to 45 years. No patients younger than 16 years (n = 152) received an opioid. Among patients with an opioid prescribed, 38 957 patients (55.5%) were female, 31 225 (47.6%) were white, and 19 194 (46.0%) were privately insured; the median age group was 46 to 50 years. Opioid prescribing peaked in 2013, both with regard to the absolute number of prescriptions (9499) and the number per patient encounter (16.3 prescriptions per 100 encounters) (Table 1; Figure 1A). Following 2013, the ED physicians prescribed notably fewer opioids. Specifically, opioid prescription rates were associated with a yearly unadjusted OR of 0.793 (95% CI, 0.787-0.799) when comparing opioid prescribing with the prior year, with 2013 being the intercept. These findings were robust to adjustment for patient age, race/ethnicity, sex, and insurance status (aOR, 0.808; 95% CI, 0.802-0.814) (Table 2). Thus, opioid prescribing decreased from 16.3 prescriptions per 100 encounters to 5.5 prescriptions per 100 encounters between 2013 and 2018—a 66.3% reduction in yearly opioid prescribing over 5 years.

**Table 1.** Demographic Characteristics of Patients Treated With an Opioid Between 2009 and 2018^a^

Characteristic	No. treated with opioids/total No. of patients (%)									
	2009	2010	2011	2012	2013	2014	2015	2016	2017	2018
Patients^b^^,^^c^	5963/50 344 (11.8)	6836/54 772 (12.5)	8445/57 482 (14.7)	9321/58 096 (16.0)	9499/58 367 (16.3)	8145/58 037 (14.0)	7808/57 438 (13.6)	6558/57 909 (11.3)	4534/56 692 (8.0)	3109/56 339 (5.5)
**Age, y**^**d**^										
16-30	222/3485 (6.4)	322/4546 (7.1)	540/5810 (9.3)	768/7001 (11.0)	971/8175 (11.9)	934/9722 (9.6)	1063/11 336 (9.4)	1044/12 935 (8.1)	774/13 731 (5.6)	549/14 683 (3.7)
31-65	4594/36 622 (12.5)	5247/39 409 (13.3)	6442/40 488 (15.9)	6919/40 484 (17.1)	6926/40 697 (17.0)	5912/39 053 (15.1)	5516/37 141 (14.8)	4532/36 321 (12.5)	3090/34 735 (8.9)	2054/33 214 (6.2)
>66	1147/9452 (12.1)	1267/9836 (12.9)	1460/10 098 (14.5)	1633/9924 (16.5)	1602/9353 (17.1)	1297/9144 (14.2)	1229/8837 (13.9)	981/8538 (11.5)	670/8104 (8.3)	506/8313 (6.1)
**Sex**										
Women	3317/28 188 (11.8)	3786/30 633 (12.4)	4709/32 125 (14.7)	5231/32 610 (16.0)	5408/32 679 (16.6)	4435/32 611 (13.6)	4354/32 285 (13.5)	3561/32 550 (10.9)	2517/31 683 (7.9)	1639/31 268 (5.2)
Men	2646/22 156 (11.9)	3050/24 139 (12.6)	3736/25 357 (14.7)	4090/25 486 (16.0)	4091/25 688 (16.6)	3710/25 426 (14.6)	3454/25 153 (13.7)	2997/25 359 (11.8)	2017/25 009 (8.1)	1470/25 071 (5.9)
**Race/ethnicity**										
White	3045/23 781 (12.8)	3036/35 845 (12.7)	3528/26 441 (13.6)	4261/26 142 (16.1)	4622/25 621 (18.0)	3920/24 352 (16.1)	2621/17 066 (15.4)	2259/17 941 (12.6)	2256/24 293 (9.4)	1677/23 588 (7.1)
Black	1677/15 593 (10.8)	1815/16 667 (10.9)	2184/17 076 (12.8)	2403/17 235 (13.9)	2431/17 766 (13.7)	2019/17 491 (11.5)	1621/14 073 (11.5)	1450/15 919 (9.1)	1214/19 829 (6.1)	734/19 624 (3.7)
Asian	129/1299 (9.9)	177/1527 (11.6)	221/1794 (12.3)	265/1894 (14.0)	226/1826 (12.38)	215/1760 (12.2)	126/1141 (11.0)	114/1373 (8.3)	117/1824 (6.4)	85/1961 (4.3)
Hispanic	633/5236 (12.1)	732/5782 (12.6)	950/6175 (15.4)	1096/6338 (17.3)	1195/6649 (17.9)	1022/6844 (14.4)	976/6765 (14.4)	855/7301 (11.7)	652/7123 (9.2)	421/7529 (5.6)
Other/NR	479/4336 (11.0)	566/4818 (11.8)	780/5740 (13.6)	899/6189 (14.5)	840/5111 (16.4)	811/6103 (13.3)	2343/17 075 (13.7)	1855/14 967 (12.4)	292/3473 (8.4)	190/3484 (5.5)
**Insurance (n = 211 722)**										
Private	1258/9975 (12.6)	1454/10 969 (13.3)	1989/12 350 (16.1)	2324/13 042 (17.8)	2544/13 615 (18.7)	2202/13 728 (16.0)	2222/14 351 (15.5)	1978/15 174 (13.0)	1539/15 848 (9.7)	1684/24 360 (6.9)
Medicare	639/4925 (13.0)	752/5631 (13.4)	904/6266 (14.4)	1093/6562 (16.7)	1080/6758 (16.0)	984/6879 (14.3)	999/7375 (13.6)	826/7585 (10.9)	605/8385 (7.2)	552/10 022 (5.5)
Medicaid	296/2649 (11.1)	344/3153 (10.9)	405/2256 (12.1)	520/3759 (13.8)	537/4273 (12.6)	534/4585 (11.7)	533/5054 (10.6)	545/6062 (9.0)	395/7061 (5.6)	390/12 070 (3.2)
Self-pay	521/4147 (12.6)	641/4848 (13.2)	779/5311 (14.7)	944/5800 (16.3)	964/6164 (15.6)	928/6842 (13.6)	1014/7625 (13.3)	1547/14 597 (10.6)	1805/22 979 (7.9)	469/9567 (4.9)

Abbreviation: NR, not reported.

^a^ Temporal opioid use within the Northwestern Memorial Hospital emergency department, including all patients seen and within demographic subgroups for 2-year increments.

^b^ Patients who were aged 31 to 65 years, white race, and privately insured demonstrated the highest proportion of encounters resulting in an opioid prescription.

^c^ Twenty of the 70 218 opioids were opioid receptor antagonists (naltrexone, naloxone, or buprenorphine).

^d^ Only 152 patients were younger than 16 years because pediatric patients are usually transported to Lurie Children's Hospital emergency department. None received an opioid.

**Table 2.** Temporal Opioid Prescription and Odds of Opioid Prescription Compared With the Prior Year by Condition

Condition	No. treated with opioids/total No. of patients (%)						aOR (95% CI)	
	2013	2014	2015	2016	2017	2018	Model 1^a^	Model 2^b^
**Musculoskeletal pain**^c^								
Back pain	1029/2129 (48.3	870/2086 (41.7)	516/1337 (38.6)	673/1803 (37.3)	468/1795 (26.1)	310/2046 (15.2)	0.744 (0.725-0.765)	0.759 (0.738-0.780)
Joint pain	526/1635 (32.2)	379/1419 (26.7)	218/796 (27.4)	289/1292 (22.4)	170/1127 (15.1)	120/1302 (9.2)	0.748 (0.722-0.776)	0.760 (0.733-0.788)
Limb pain	213/1039 (20.5)	151/901 (16.8)	90/557 (16.2)	130/1016 (12.8)	72/957 (7.2)	54/1106 (4.9)	0.718 (0.682-0.756)	0.729 (0.691-0.768)
Neck pain	127/361 (35.2)	81/291 (27.8)	55/188 (29.3)	71/347 (20.5)	36/348 (10.3)	20/312 (6.4)	0.681 (0.631-0.736)	0.687 (0.635-0.744)
Musculoskeletal pain	1895/5164 (36.7)	1895/4697 (36.7)	879/2878 (30.5)	879/4458 (30.5)	746/4277 (17.7)	504/4764 (10.6)	0.746 (0.732-0.760)	0.758 (0.744-0.773)
**Musculoskeletal trauma**								
Fracture	1168/1903 (61.4)	1112/2017 (55.1)	721/1314 (54.9)	1001/2130 (47.0)	856/2093 (40.9)	680/2051 (33.2)	0.802 (0.781-0.823)	0.809 (0.788-0.832)
Sprain	525/1549 (24.0)	412/1473 (16.8)	210/836 (15.6)	178/928 (15.1)	91/761 (8.6)	77/1045 (5.0)	0.702 (0.672-0.733)	0.762 (0.719-0.808)
Contusion	283/1180 (33.9)	196/1169 (28.0)	106/681 (25.1)	138/914 (19.2)	61/709 (12.0)	39/786 (7.4)	0.744 (0.703-0.787)	0.706 (0.675-0.738)
Other injury	821/3314 (24.8)	635/3248 (19.6)	389/2229 (17.5)	398/3324 (12.0)	284/3361 (8.4)	135/2529 (5.3)	0.706 (0.685-0.729)	0.711 (0.688-0.734)
Musculoskeletal trauma	2782/7946 (34.2)	2355/7907 (29.8)	1415/5035 (28.1)	1672/7149 (23.4)	1235/6733 (18.3)	872/5910 (14.75)	0.799 (0.786-0.812)	0.811 (0.797-0.824)
**Other pain**								
Abdominal pain	794/3861 (20.6)	721/4005 (18.0)	443/2615 (16.9)	408/3060 (13.3)	268/2962 (9.0)	212/3458 (6.1)	0.769 (0.749-0.790)	0.789 (0.767-0.811)
Kidney stone	346/494 (70.0)	403/587 (68.7)	286/417 (68.6)	429/704 (60.9)	365/628 (58.1)	423/814 (52.0)	0.858 (0.819-0.899)	0.855 (0.816-0.897)
Respiratory distress	198/2474 (8.0)	153/2448 (6.3)	101/1695 (6.0)	96/2343 (4.1)	43/2194 (2.0)	35/2701 (1.3)	0.698 (0.641-0.760)	0.716 (0.656-0.782)
Pharyngitis	134/500 (26.8)	77/442 (17.4)	69/308 (22.4)	45/262 (17.2)	29/211 (13.7)	13/336 (3.9)	0.697 (0.659-0.738)	0.709 (0.669-0.750)
All other pain	1472/7329 (20.1)	1354/7481 (18.1)	899/5035 (17.9)	978/6470 (15.1)	705/6095 (11.6)	683/7309 (9.3)	0.840 (0.825-0.855)	0.850 (0.834-0.866)
**All ED**								
Patients	9499/58 367 (16.3)	8145/58 037 (14.0)	7808/57 438 (13.6)	6558/57 909 (11.3)	4534/56 692 (8.0)	3109/56 339 (5.5)	0.793 (0.787-0.799)	0.808 (0.802-0.814)

Abbreviation: aOR, adjusted odds ratio.

^a^ Logistic regression of opioid use (yes or no) on year.

^b^ Logistic regression of opioid use (yes or no) on year controlling for patient age, sex, race/ethnicity, and insurance status.

^c^ Musculoskeletal pain demonstrated the greatest decrease in opioid use, beyond the decrease seen for all patients within the department. The yearly decrease was more significant than the yearly decrease for the entire emergency department, even when controlling for patient demographic characteristics.

Generally, musculoskeletal pain conditions (back, joint, limb, and neck pain) were associated with the greatest proportional decrease in opioid prescribing from 2013 to 2018 (71.1% decrease: from 36.7 to 10.6 per 100 patients; OR, 0.746; 95% CI, 0.732-0.760; aOR, 0.758; 95% CI, 0.744-0.773), followed by musculoskeletal trauma (fracture, sprain, contusion, and injury) (58.0% decrease: from 35.2 to 14.8 per 100 patients; OR, 0.799; 95% CI, 0.786-0.812; aOR, 0.811; 95% CI, 0.797-0.824) and nonmusculoskeletal pain (abdominal pain, kidney stone, respiratory distress, and pharyngitis) (53.7% decrease: from 20.1 to 9.3 per 100 patients; OR, 0.840; 95% CI, 0.825-0.855; aOR, 0.850; 95% CI, 0.834-0.868). Some heterogeneity was present within these groups of conditions. For instance, musculoskeletal pain conditions decreased between 68.5% (back pain) and 81.8% (neck pain) between 2013 and 2018. Musculoskeletal trauma conditions decreased between 45.9% (fracture) and 76.6% (sprains). Conversely, prescriptions decreased in patients with kidney stones by only 25.7% between 2013 and 2018. These differential decreases in opioid prescribing are depicted in Figure 1, which presents the more significant reduction in opioids prescribed in musculoskeletal pain compared with all patients seen in the ED, musculoskeletal trauma conditions, and nonmusculoskeletal pain conditions.

**Figure 2.**
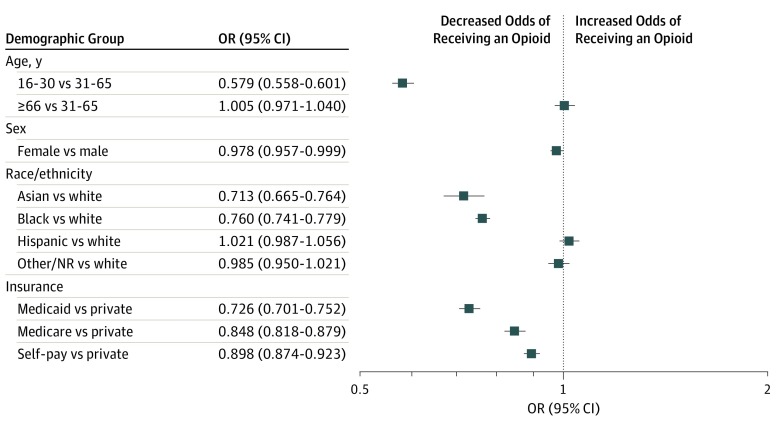
Opioid Prescribing Within Demographic Groups Between 2009 and 2018 NR indicates not reported; OR, odds ratio.

Across all years, compared with their demographic counterparts, patients who were black (aOR, 0.760; 95% CI, 0.741-0.779), Asian (aOR, 0.714; 95% CI, 0.665-0.764), receiving Medicaid (aOR, 0.726; 95% CI, 0.701-0.752), and aged 16 to 30 years (aOR, 0.579; 95% CI, 0.558-0.601) had the lowest odds of receiving an opioid for treatment of pain. Differences in opioid prescribing for female and male patients were minimal (Figure 2). In addition, across all age, race/ethnicity, sex, and insurance status groups, opioid prescribing decreased from 2013 to 2018 (Table 1). With regard to insurance status, patients with Medicaid had the greatest yearly decrease (aOR, 0.766; 95% CI, 0.750-0.782) of opioid prescriptions; privately insured patients were the only subgroup associated with a less substantial yearly decrease than the overall ED population (aOR, 0.848; 95% CI, 0.841-0.855). Examining differences among race showed an association between black race and the greatest yearly decrease (aOR, 0.784; 95% CI, 0.772-0.797) after 2013. The decrease in opioid prescription between male (OR, 0.803; 95% CI, 0.796-0.810) and female (OR, 0.814; 95% CI, 0.805-0.823) patients showed no distinction (eFigure in the Supplement).

The peak opioid prescription rates for each clinician in any single year between 2012 and 2015 ranged from 15.1 to 19.9 opioid prescriptions per 100 encounters. All physicians decreased the number of opioid prescriptions, such that in 2017, no single physician of the 12 included in the analysis prescribed more than 8.8 opioids per 100 encounters, which was associated with a 44.7% to 61.9% decrease from 2013 to 2017. The decrease in opioid prescribing was substantial and relatively similar in magnitude across 11 of 12 clinicians when controlling for patient demographic characteristics (Table 3).

**Table 3.** Temporal Opioid Use and Odds of Opioid Use Compared With the Prior Year for Individual Clinicians^a^

Clinician	No. treated with opioids/total No. of patients (%)					aOR (95% CI)^b^
	2013	2014	2015	2016	2017	
1	333/1892 (17.6)	368/2274 (16.2)	282/2052 (13.7)	327/2325 (14.06)	210/2581 (8.1)	0.836 (0.797-0.877)
2	238/1806 (13.2)	265/2216 (12.0)	281/2165 (13.0)	204/2758 (11.0)	186/2683 (7.3)	0.877 (0.834-0.923)
3	296/1786 (16.6)	305/2366 (12.9)	260/2114 (12.3)	257/2472 (10.4)	137/1662 (8.2)	0.824 (0.779-0.871)
4	251/1658 (15.1)	446/3241 (13.8)	430/3293 (13.0)	378/4109 (9.2)	268/4302 (6.2)	0.800 (0.763-0.838)
5	202/1260 (16.0)	231/1566 (14.8)	279/1831 (15.2)	280/2095 (13.4)	165/1879 (8.8)	0.865 (0.819-0.914)
6	213/1449 (14.7)	219/1514 (14.5)	219/1578 (13.9)	198/1615 (12.3)	130/1831 (7.1)	0.856 (0.806-0.909)
7	191/1220 (16.7)	212/1390 (15.3)	212/1387 (12.8)	163/1482 (11.0)	133/1505 (8.8)	0.862 (0.810-0.918)
8	0/2 (0)	138/706 (19.6)	235/1654 (14.2)	195/1924 (10.1)	140/1818 (7.7)	0.700 (0.638-0.769)
9	364/2085 (17.5)	308/2227 (13.8)	269/1932 (13.9)	238/1892 (12.6)	171/1936 (8.83)	0.884 (0.840-0.930)
10	164/1126 (14.6)	182/1375 (13.2)	147/1258 (11.7)	143/1459 (9.8)	107/1393 (7.7)	0.843 (0.788-0.902)
11	234/1384 (16.9)	286/1758 (16.3)	264/1597 (16.5)	194/1506 (12.9)	132/1634 (8.1)	0.822 (0.778-0.870)
12	157/921 (17.1)	159/1413 (11.3)	165/1280 (12.9)	120/1391 (8.6)	74/1146 (6.5)	0.806 (0.748-0.869)

Abbreviation: aOR, adjusted odds ratio.

^a^ When controlling for patient demographic characteristics, all clinicians demonstrated significant decreases in opioid use; 95% CIs demonstrate that these changes occurred with relatively equal magnitude for 11 of the 12 clinicians.

^b^ Logistic regression of opioid (yes or no) on year, controlling for patient age, sex, race/ethnicity, and insurance status.

## Discussion

Much attention has been given to the prescribing of opioids for pain by US physicians in response to the opioid epidemic. In a study of opioid prescribing within an urban academic ED, our analysis notes the expected temporal changes given the nationwide attention to opioid prescribing while providing details of prescription patterns by physicians for patients within certain conditions and demographic subgroups over time. From 2013 to 2018, the ED experienced a 66.3% decrease in opioid prescriptions—a much greater reduction than the national decrease of 22% from 2013 to 2017.^20^ This reduction exceeds the 54% decrease in initial treatment in nationwide opioid prescribing for opioid-naive patients and is markedly greater than the 16% decrease for all patients (naive and non-naive) reported in a recent study.^22^

Although opioid prescribing for patients with all conditions evaluated decreased from 2013 to 2018, the magnitude of decrease was, to a major extent, associated with large decreases for patients with musculoskeletal pain. Reduction in opioid treatment of musculoskeletal pain conditions decreased by 71.1% (from 36.7 to 10.6 per 100 patients from 2013 to 2018), which is a more substantial rate of reduction than the overall ED rate of 66.3% over the same period. This decrease was not noted for patients with a musculoskeletal trauma diagnosis (58.0% decrease from 35.2 to 14.8 per 100 patients) or patients with a nonmusculoskeletal pain diagnosis (53.7% decrease from 20.1 to 9.3 per 100 patients) over the same period. This substantial reduction in opioid prescriptions for musculoskeletal pain conditions may be due to the understanding that opioids used for the treatment of musculoskeletal pain have minimal effect on pain and disability,^25^ high opioid burden,^17,26,27^ increased adverse effects,^28^ and possible increased likelihood of repeated use from a single opioid prescription.^6,29^ Likewise, many of the patients diagnosed with back, joint, limb, and neck pain have this pain chronically and present to the ED for acute pain episodes with regularity.^17,30,31^ Guidelines recommend against opioid prescription in these cases.^21^ With up to 10% to 16% of patients presenting to the ED with chronic pain,^32,33^ these musculoskeletal conditions are an important diagnostic group to target for nonopioid pharmacologic interventions.

All patient demographic subgroups saw a decrease in opioids prescribed for them following the peak of opioid prescribing in 2013. Comparing racial subgroups, black race was associated with the greatest decrease in opioid prescribing, as well as the lowest odds of receiving opioids across the entire decade. This finding is consistent with data reporting lower doses of analgesics provided to patients of minority racial/ethnic groups predating the recognition of the opioid crisis, as opposed to white patients who have historically had the highest likelihood of receiving opioids.^34,35^ Patients with Medicaid had the lowest odds of receiving an opioid—a group in which a prior study noted a high burden of opioid prescriptions in the ED for acute pain.^27^ In terms of patient age and in contrast to a nationwide study of ED opioid prescribing, there was no statistically significant difference in opioid prescribing between patients aged 31 to 65 and older than 65 years.^36^

At an individual clinician level, all analyzed physicians were associated with markedly and similarly reduced prescription rates from 2013 to 2017. A recent study showed an association between guidelines and prescribing practices,^37^ and during the decrease of opioid prescribing in the ED in the present study, guidelines from the Illinois Drug Monitoring Program,^38^ the Centers for Disease Control and Prevention, the surgeon general, and throughout emergency medicine literature were published.^19,21,39,40,41,42^ Furthermore, in 2017, a quality-control program was implemented within our ED, in which quarterly prescribing patterns were reviewed by the individual clinicians who were compared with their peers.^43^ The consistency of reduction in opioid prescribing among clinicians demonstrates that treatment decisions are made not only on an individual level, but also within the larger context of the medical environment in which physicians are influenced by guidelines and departmental policy.

### Limitations

This study has several limitations. The use of *ICD* codes for conditions does not necessarily mean the patient was given the opioid for that condition, although steps were made to diminish this possible factor. As always, a drug prescribed for a patient does not guarantee consumption. Pharmacotherapy using nonopioid alternatives does not necessarily improve an individual outcome, and given that this study was conducted in an ED, long-term outcomes (repeat visits, repeat prescriptions, and opioid use disorder) are difficult to analyze. Data on the severity of pain were not available and comorbidities (eg, cancer) were not analyzed, although this information likely would not change the overall conclusion. In addition, the change from *ICD-9* to *ICD-10* diagnosis codes in 2015 created discrepancies between the number of patients in that year compared with the other years, so caution should be used in examining 2015 data independently from the overall pattern during the study period. Another limitation is that this study did not have robust data for quantity and dose of the opioid used—this information is important because higher morphine milligram equivalents are associated with long-term opioid use and death,^44,45,46^ and the clinician analyses in prior studies included this factor to define high- and low-intensity prescribing patterns in clinicians.^5,22^ These data points were intermittent owing to interruptions in data collection at the Enterprise Data Warehouse from various electronic health record changes. In addition, we recognize that the generalizability of this study, given that it focuses on a single department with a single set of physicians, is limited. This study reports, however, an association between a targeted reduction in opioid prescriptions for musculoskeletal pain conditions, such as back, joint, limb, and neck pain, and a major decrease in opioid prescribing, including a collective decrease in opioid prescriptions across all clinicians within the ED.

## Conclusions

The goal in the pharmacotherapy of pain relief is to use the drugs available as appropriately as possible. Although opioids are effective and may still have a place in treating severely painful conditions with a self-limited, short-term time course, studies have indicated that nonsteroidal anti-inflammatory drugs are also effective in treating certain pain.^11,47,48,49^ As noted in this study, the greatest reduction in opioid prescribing was for musculoskeletal pain disorders, and a smaller reduction was seen in musculoskeletal trauma and nonmusculoskeletal pain conditions, most notably kidney stones. Although it is difficult to discern whether the number of opioid prescriptions inherently reduces the risk of repeated use of opioids or opioid use disorder, there is an association between single ED opioid prescriptions leading to long-term use of opioids,^6,7^ and the ED accounts for over 20% of nationwide number of opioid prescriptions.^50^ This study suggests that substantial relative decreases in opioids for treatment of back, joint, limb, and neck pain allow for selective prescribing of opioids for treatment of acute, self-limited pain seen with musculoskeletal trauma and kidney stones, while continuing to reduce overall opioid prescribing within an ED. Studies should continue to elucidate situations in which opioid and nonopioid analgesic therapy is indicated and associated with good clinical outcomes.

## Notes

Supplement. **eTable 1.** Opioid Selection and Prescription Numbers **eTable 2.** ICD Code Selection **eFigure.** Yearly Decline in Opioid Prescription Within Demographic Subgroups After 2013
