# Supplementary material for: Temporal Factors Associated With Opioid Prescriptions for Patients With Pain Conditions in an Urban Emergency Department
Source: JAMA Netw Open. 2020 Mar 25;3(3):e200802. doi: 10.1001/jamanetworkopen.2020.0802 (PMC7097712; doi:10.1001/jamanetworkopen.2020.0802)
Supplement: Supplement. — eTable 1. Opioid Selection and Prescription Numbers eTable 2. ICD Code Selection eFigure. Yearly Decline in Opioid Prescription Within Demographic Subgroups After 2013 [file jamanetwopen-3-e200802-s001.pdf]

Supplementary Online Content

Smith BC, Vigotsky AD, Apkarian AV, Schnitzer TJ. Temporal factors associated with opioid prescriptions for patients with pain conditions in an urban emergency department. *JAMA Netw Open*. 2020;3(3):e200802. doi:10.1001/jamanetworkopen.2020.0802

eTable 1. Opioid Selection and Prescription Numbers

eTable 2. ICD Code Selection

eFigure. Yearly Decline in Opioid Prescription Within Demographic Subgroups After 2013

This supplementary material has been provided by the authors to give readers additional information about their work.

.

© 2020 Smith BC et al. *JAMA Network Open.*

eTable 1: Opioid Selection and Prescription Numbers		
Opioid	Number	Percentage
hydrocodone-acetaminophen	68,219	97.15
acetaminophen-codeine	644	0.92
hydromorphone	408	0.58
acetaminophen-oxycodone	291	0.41
oxycodone	247	0.35
morphine	181	0.26
hydrocodone-ibuprofen	87	0.12
fentanyl	51	0.07
acetaminophen-propoxyphene	22	0.03
acetaminophen-tramadol	20	0.03
methadone	16	0.02
naloxone	8	0.01
bupropion-naloxone	7	0.01
methylnaltrexone	3	<0.01
naltrexone	3	<0.01
tramadol	3	<0.01
tapentadol	2	<0.01
hydrocodone	1	<0.01
aspirin-codeine	1	<0.01
aspirin oxycodone	1	<0.01
codeine-sulfate	1	<0.01
ibuprofen-oxycodone	1	<0.01
opium	1	<0.01

© 2020 Smith BC et al. *JAMA Network Open.*

eTable 2: ICD Code Selection		
Diagnosis	ICD-9-CM	ICD-10-CM
Back Pain	724.x	M53.9, M54.x
Joint Pain	719.4	M25.5
Limb Pain	729.5	M79.6
Neck Pain	723.1	M54.2
Fracture	800-829	Sx2
Sprain	840-849.x	Sx3
Contusion	920-924.x	Sx0, S05
Other Injury	959.x	Sx9, T14
Abdominal Pain	789.0, 789.6	R10
Kidney Stone	592.0, 592.1, 592.9	N20.0, N20.9
Resp. Distress	786.5	R07
Pharyngitis	462	J02.9

© 2020 Smith BC et al. *JAMA Network Open.*

eFigure: Yearly Decline in Opioid Prescription within Demographic Subgroups after 2013

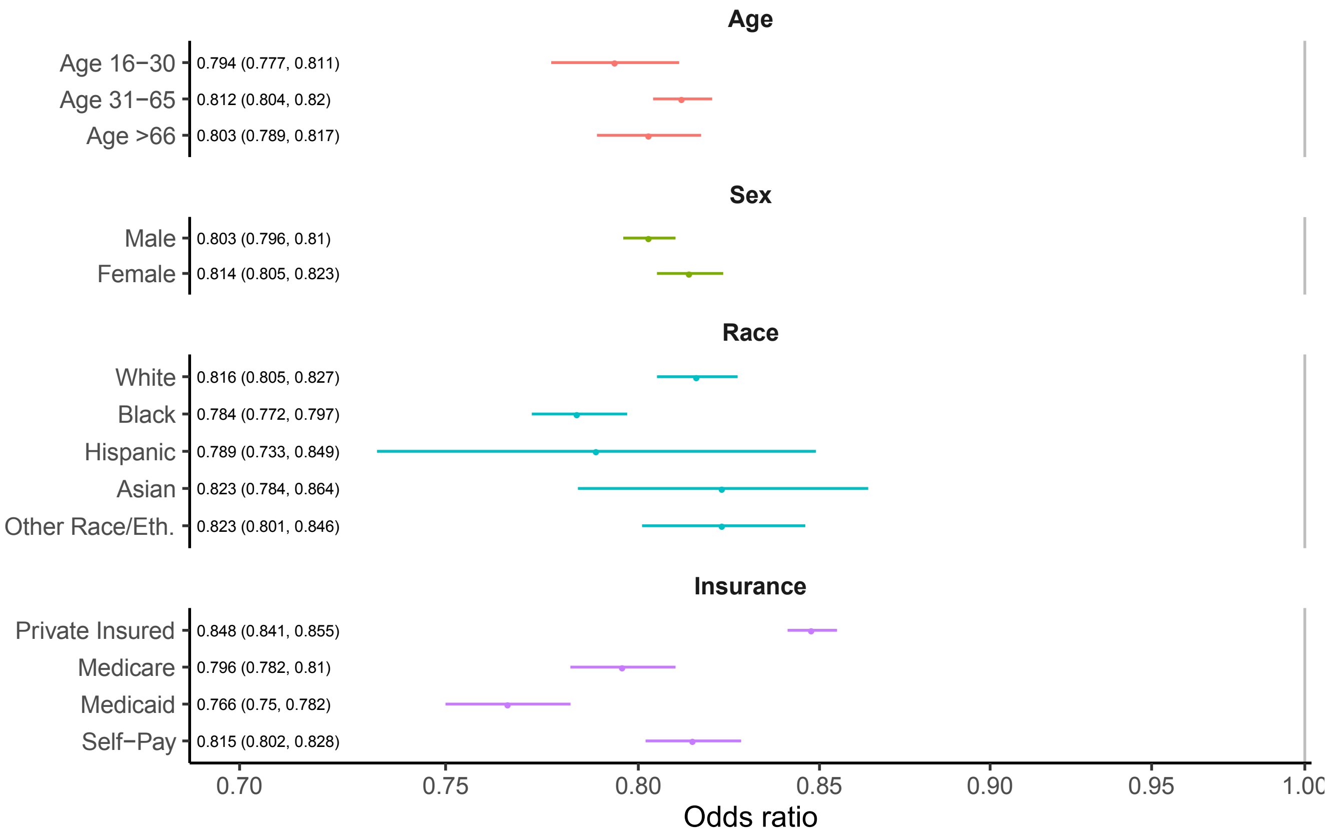

All demographic subgroups were associated with a significant decline in yearly opioid prescriptions after 2013.

© 2020 Smith BC et al. *JAMA Network Open.*
